# The New Infirmary at Bristol

**Published:** 1901-03-16

**Authors:** 


### THE NEW INFIRMARY AT BRISTOL.

As an apt illustration of some of the difficulties attending architectural competitions for hospital plans to which we alluded in a recent article, we publish the following award made by the Assessor appointed by the Board of Guardians at Bristol to examine the plans sent in for the new Poor-law Infirmary in that city.

It will be noted that he at once states that the estimates given by all the competitors appear to him to be "more or less inadequate," and, what is worse, that "the figures on which these estimates are based appear in each case inaccurately stated," and one cannot but feel that the meaning of the award is that the Guardians by tying the competitors down to an impossible limit as to cost have brought upon themselves a series of fictitious estimates which they will sooner or later have much cause to regret. When we see a plan which is estimated by the architect to cost £115,504 jump up at once, under the criticism of the Assessor, to a sum of £191,867, we can understand what a farce a competition may become if impossible conditions and impossible prices are made to form part of the conditions, for the Assessor says that even these estimates must not be taken as representing what such a hospital as the Guardians require would in his opinion cost. That the competing architects should have sent in estimates "which they ought to have known are hopelessly inadequate" is bad enough, although we are not quite sure that the whole system of the competition, and the limitations under which it was carried out, are not even more strongly condemned by the fact that the Assessor found himself driven to place first on the list a design which contains many features which he expects the Local Government Board (with whom the final decision rests) would hesitate to accept or approve; a design moreover, the report submitted, containing paragraphs which, in his opinion, "constitute a very grave departure from the recognised rules governing every competition on which the strictest anonymity is intended to be preserved."

### Assessor's Award on the Plans.

17 Southampton Street, Bloomsbury,
London, W.C.,
December 29th, 1900.

Gentlemen,—In accordance with your instructions, I have carefully examined and compared the seven sets of designs submitted in competition for your proposed New Infirmary, and beg to report as follows:—

The three designs which best carry out the instructions, and to which I consider the first three places should be awarded, are—

1 ... ... ... ... E.
2 ... ... ... ... A.
3 ... ... ... ... B.

In making this award I have considered the comparative merits of each set of plans, giving due weight also to the question of cost.

The estimates given by these competitors appear to be all more or less inadequate, and the figures on which these estimates are based appear in each case inaccurately stated, though not equally so.

The following are the competitors' estimates:—

| | General Buildings. | | Laundry and Kitchen Fittings. | | Total. |
|---|---|---|---|---|---|
| | £ | | £ | | £ |
| E. ... | 123,383 | ... | 2,200 | ... | 125,583. |
| A. ... | 109,004 | ... | 6,500 | ... | 115,504. |
| B. ... | 127,338 | ... | 2,000 | ... | 129,338. |

Of these, the amount given by A. for laundry and kitchen fittings is, in my opinion, a more reasonable one than either of the others.

After correcting the errors in cubing and in calculation in all the three estimates under consideration, I think the following amounts may be taken to represent approximately the comparative money values of the three designs:—

| | £ |
|---|---|
| E. ... ... ... ... | 153,796 |
| A. ... ... ... ... | 191,869 |
| B. ... ... ... ... | 166,384 |

It must be understood, however, that these estimates are, as stated, only comparative, and do not represent what such a hospital as the guardians require, if properly designed in all its parts, would in my opinion cost.

In the foregoing estimates each design is priced at the same figure per foot cube, and I have assumed that there would be no material difference in the quality of the work to justify any difference in the price.

It is clear to my mind that the limit of cost proposed by the guardians is very far from being sufficient to provide the accommodation required; it would have been better if this fact, which must have been obvious to the competitors, had been frankly pointed out by them. They have, however, preferred to send in estimates which they ought to have known are hopelessly inadequate.

With regard to the general question of design, while I consider E. design to be unquestionably superior in all important points to the two others, I am bound to point out that there are many features in the arrangement which I should expect the Local Government Board would hesitate to accept or approve.

While placing design E. first in order of merit, I cannot but refer to some paragraphs in the report submitted with that design which constitute, in my opinion, a very grave departure from the recognised rules governing every competition on which the strictest anonymity is intended to be preserved. The paragraphs I refer to are the second on page 1, the third on page 9, the second on page 19, and the paragraph in brackets on page 20. While I cannot say that the statements referred to constitute a breach of the conditions, I think it is much to be regretted that any competitor should have endeavoured to advertise his special experience in this class of work in the way, as it appears to me, the authors of this design have done. I am, gentlemen,

Your obedient servant,

KEITH D. YOUNG.

To the Chairman and Members of the Board of Guardians of the City and County of Bristol.

